# Volatile Metabolite Profiling of Durum Wheat Kernels Contaminated by *Fusarium poae*

**DOI:** 10.3390/metabo4040932

**Published:** 2014-10-17

**Authors:** Barbara Laddomada, Laura Del Coco, Miriana Durante, Dominique S. Presicce, Pietro A. Siciliano, Francesco P. Fanizzi, Antonio F. Logrieco

**Affiliations:** 1 C.N.R. Institute of Sciences of Food Production (ISPA), Prov.le Lecce-Monteroni, 73100 Lecce, Italy; E-Mails: barbara.laddomada@ispa.cnr.it (B.L.); miriana.durante@ispa.cnr.it (M.D.); 2 Di.S.Te.B.A., University of Salento, Prov.le Lecce-Monteroni, 73100 Lecce, Italy; E-Mail: laura.delcoco@unisalento.it; 3 C.N.R. Institute for Microelectronics and Microsystems (IMM), Prov.le Lecce-Monteroni, 73100 Lecce, Italy; E-Mails: Dominique.presicce@gmail.com (D.S.P.); pietro.siciliano@le.imm.cnr.it (P.A.S.); 4 C.N.R. Institute of Sciences of Food Production (ISPA), Via Amendola 122/O, 70125 Bari, Italy

**Keywords:** *Fusarium poae*, durum wheat, volatile metabolites, SPME-GC/MS, PCA

## Abstract

Volatile metabolites from mold contamination have been proposed for the early identification of toxigenic fungi to prevent toxicological risks, but there are no such data available for *Fusarium poae.* *F. poae* is one of the species complexes involved in *Fusarium* head blight, a cereal disease that results in significant yield losses and quality reductions. The identification of volatile organic compounds associated with *F. poae* metabolism could provide good markers to indicate early fungal contamination. To this aim, we evaluated the volatile profile of healthy and *F. poae*-infected durum wheat kernels by SPME-GC/MS analysis. The production of volatile metabolites was monitored for seven days, and the time course analysis of key volatiles was determined. A total of 29 volatile markers were selected among the detected compounds, and multivariate analysis was applied to establish the relationship between potential volatile markers and fungal contamination. A range of volatile compounds, including alcohols, ketones, esters, furans and aromatics, were identified, both in contaminated and in healthy kernels. However, the overall volatile profile of infected samples and controls differed, indicating that the whole volatile profile, rather than individual volatile compounds, could be used to identify *F. poae* contamination of durum wheat grains.

## 1. Introduction

*Fusarium* head blight (FHB) is a significant disease of wheat, barley and small grain cereals causing high yield loss worldwide. Among the *Fusarium* species responsible for FHB, *F. graminearum* is considered the most important, though *F. avenaceum*, *F. culmorum* and *F. poae* are also involved as FHB components, especially in North Europe and North America [1,2,3]. The distribution and predominance of the FHB pathogens is, to a large extent, determined by climatic factors, particularly temperature and moisture [4,5].

The epidemiology and biology of *F. poae* have been studied in depth, with particular regard to the production of mycotoxins [6,7]. The mycotoxins produced by *F. poae* include type-A and type-B trichothecenes, such as diacetoxyscirpenol (DAS), monoacetoxycirpenols (MAS), scirpentriol (SCR), nivalenol (NIV) and fusarenon-X (FX) [8,9,10,11,12]. Reports concerning the ability of *F. poae* isolates to produce HT-2 and T-2 toxins and neosolaniol continue to be contradictory [10,11,13], probably due to the misidentification of *F. langsethiae* isolates, which are morphologically similar to *F. poae* [10,14,15]. As a result, the contamination of cereal grains by *F. poae* may lead to significant quality decline in cereal-based food products [8]. Actually, *F. poae* toxins can be stable under normal processing conditions, causing concrete damage in consumers due to their neurotoxic, carcinogenic and cytotoxic activities [16,17].

Due to the above evidence, the European Community set maximum limits of mycotoxins in food and feed products to prevent toxicological risks for consumers [18], and the detection of *F. poae* on cereal grains and on cereal-based foods has become an important task to prevent potential toxicological risks. A range of studies have shown that volatile compounds can allow the rapid and early detection of mycotoxigenic species contaminating cereal grains [19]. Filamentous fungi, such as *F. sporotrichioides*, *Penicillium verrucosum* and *F. culmorum*, produce a number of distinctive volatiles that have been proposed as contamination markers [20,21,22,23]. In fact, *Fusarium* species contaminating cereals are able to digest the proteins and starch of infected grains [24]. Thus, a mixture of specific volatiles may arise from *Fusarium* contamination, depending on fungal species, environmental and physiological conditions [25]. Although the emission of volatiles from fungal-infected cereal grains has been extensively studied over the last few decades [19,23], to our knowledge, there are no reports on volatile compounds related to *F. poae* contamination of wheat grains [26,27,28].

Our aims were to assess the volatiles produced by *F. poae* ITEM 3258 on durum wheat grains by SPME-GC/MS in order to identify potential key volatiles to be used as markers of early contamination.

## 2. Results and Discussion

**Figure 1:**
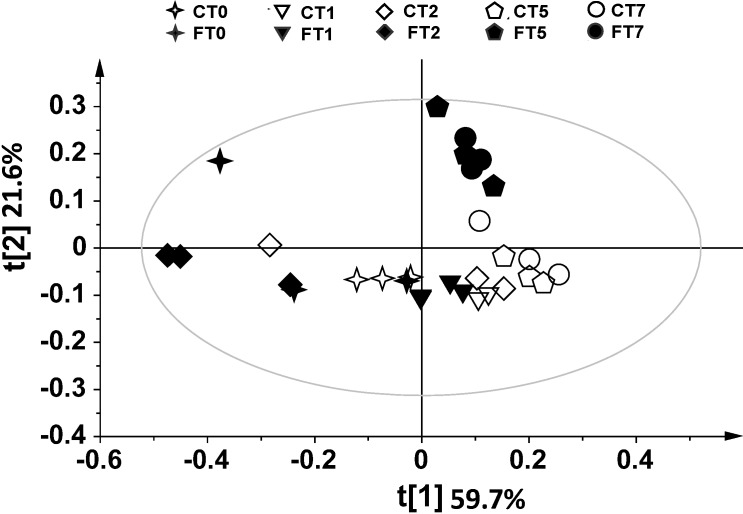
PCA t[1]/t[2] score plot of SPME-GC/MS data. FT0, FT1, FT2, FT5 and FT7: *F. poae*-infected samples, analyzed at Day 0, 1, 2, 5 and 7, respectively; CT0, CT1, CT2, CT5 and CT7: controls analyzed at Day 0, 1, 2, 5 and 7, respectively. Filled symbols: *F. poae*-infected samples (FT); empty symbols: controls (CT).

**Figure 2:**
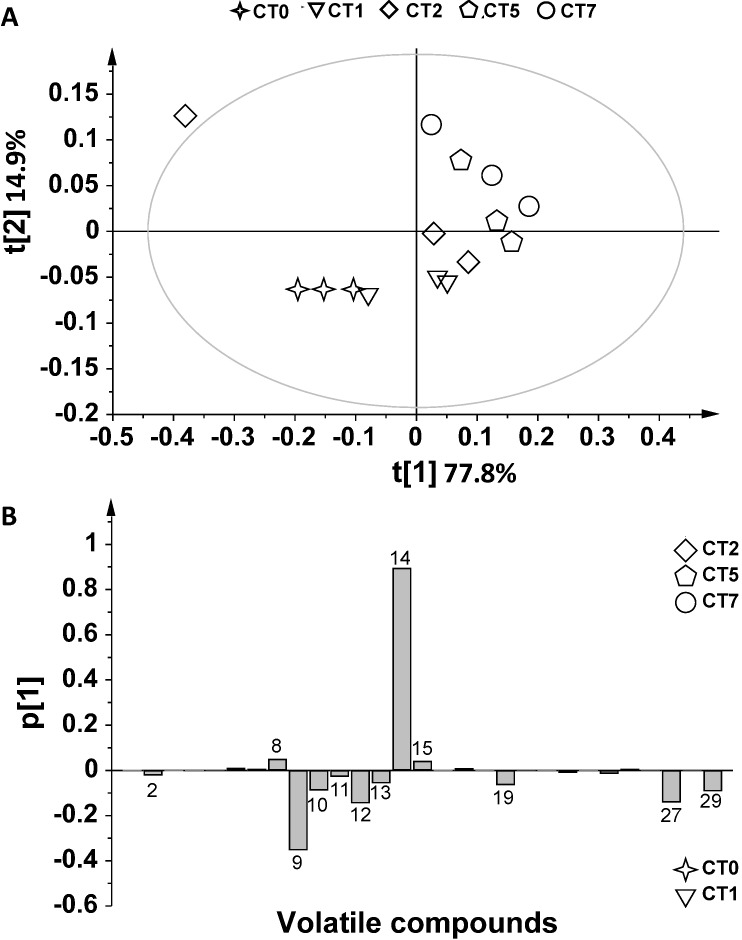
(**A**) PCA t[1]/t[2] score plot of SPME-GC/MS of CT samples analyzed at Days 0, 1, 2, 5 and 7; (**B**) p[1] loading plot for the t[1]/t[2] score plot of PCA. Positive values: metabolites detected at Days 5 (CT5) and 7 (CT7); negative values: metabolites detected at Days 0 (CT0) and 1 (CT1).

**Figure 3:**
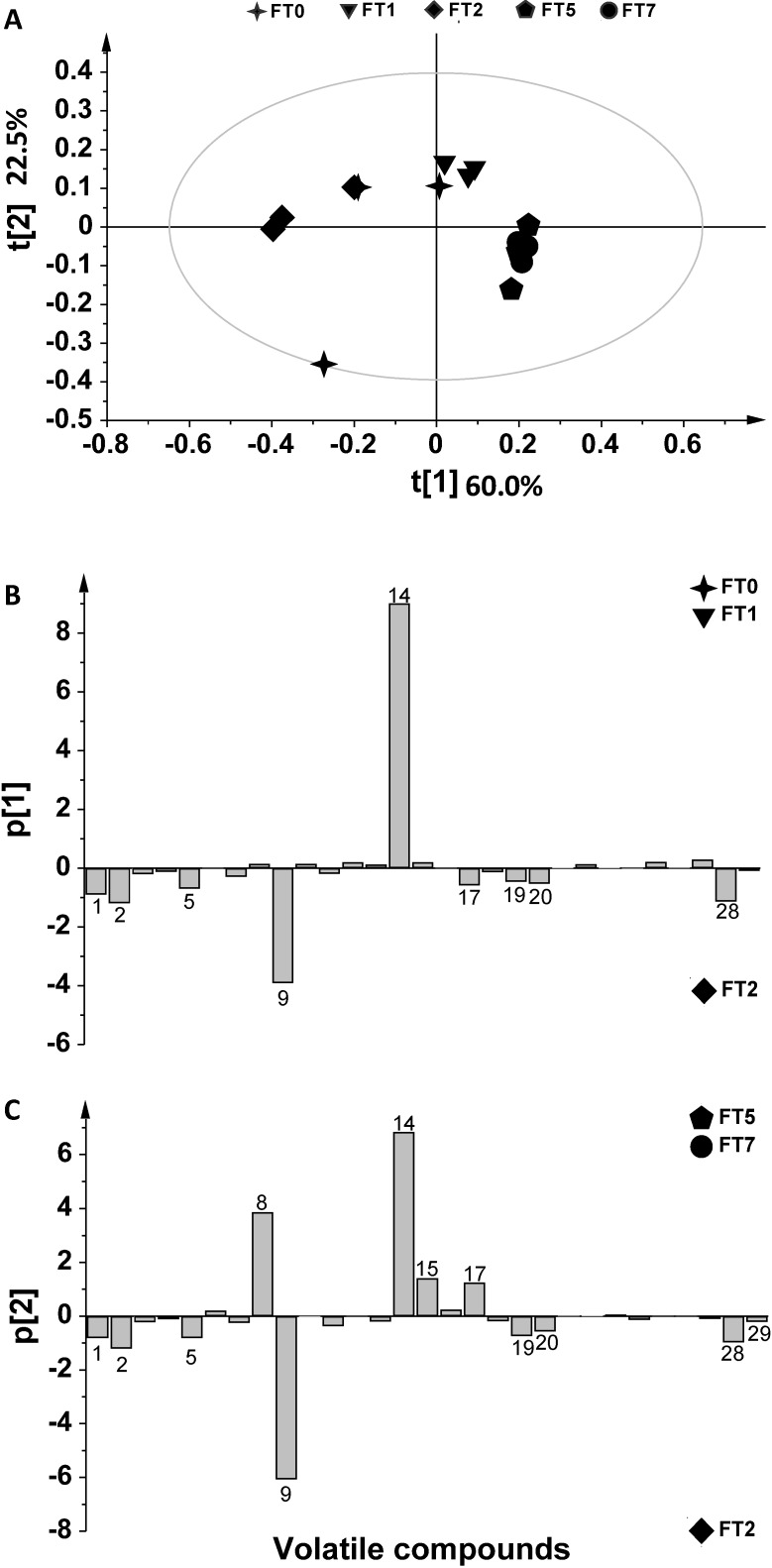
(**A**) PCA t[1]/t[2]score plot of SPME-GC/MS of FT samples analyzed at Days 0, 1, 2, 5 and 7. Filled symbols: FT samples. (**B**) p1 loading contribution plots related to the t[1]/t[2] PCA score plot. Positive values indicate metabolites identified at Days 0 (FT0), 1 (FT1); negative values refer to metabolites detected at Day 2 (FT2). (**C**) p2 loading contribution plots related to the t[1]/t[2] PCA score plot. Positive values indicate metabolites identified at Days 5 (FT5) and 7 (FT7); negative values refer to metabolites detected at Day 2 (FT2).

The volatile metabolites from infected and non-infected durum wheat grains were analyzed by SPME-GC/MS. The assays were carried out on *F. poae*-infected kernels at 0, 1, 2, 5 and 7 days after inoculation (FT0, FT1, FT2, FT5 and FT7). Non-contaminated samples (negative controls) were analyzed on the same days (CT0, CT1, CT2, CT5 and CT7) to consider the formation of volatiles normally occurring during grain aging processes. A total of 29 compounds were identified (Table 1), and a multivariate analysis was applied to establish the relationship between volatile profiles and *F. poae* contamination. The outcome data revealed that the volatile profile of contaminated grains differed from controls, as revealed by principal component analysis (PCA). The SPME-GC/MS data were first analyzed by PCA to investigate the distribution of samples and to identify potential outliers. PCA allowed the remapping of the original dataset in a new multivariate coordinate space, in which the dimensions were ordered by the decreasing explained variance in the data. The first two principal components (t[1] and t[2], as defined in the experimental section (paragraph 3.4), highlighted a large variation for volatile production among the samples. PCA of controls (CT) and of contaminated samples (FT) showed that a very useful model of the data was obtained by using t[1] and t[2] components. The score plots (t[1]/ t[2]) showed a clear separation between contaminated samples and controls starting from Day 2 (Figure 1). The explained variance of the dataset related to the two first components was 81.2%, namely 59.7% accounting for t[1], and 21.6% for t[2] (R^2^ 0.812, Q^2^ 0.258). This approach was used to obtain a general overview of the data distribution. For a better understanding of metabolic profiles in non-infected conditions, the controls were analyzed separately from FT samples, and a multivariate statistical analysis was applied (Figure 2A). The t[1] direction of PCA highlighted a linear growth from negative to positive values, with the exception of one sample (CT2, symbol ◊). The explained variance of the dataset related to the first two components was 92.7%, namely 77.8% for t[1] and 14.9% for t[2] (R^2^ 0.927, Q^2^ 0.457). The score plot provided in Figure 2B shows a more detailed t[1] direction interpretation of the scores. In fact, the samples located on the negative side of the score plot resulted in negative loadings in the line-plot p[1] (see paragraph 3.4 in the experimental section), whereas samples located on the positive side of the score plot had positive p[1] loadings in the line-plot. Thus, ethyl acetate, ethanol and 3-methylbutanol levels (*n*. 8, 14, 15, respectively) increased over time, whereas ethyl decanoate, ethyl decenoate, 2-phenylethyl acetate, 3-methylbutanal, hexanal, phenylethyl alcohol, 3-hydroxy-2-butanone and acetic acid levels (*n*. 9, 10, 11, 12, 13, 19, 27 and 29) decreased. The infected samples were then analyzed separately (Figure 3A). The explained variance of the dataset for the two first components was 82.5%, namely 60% for t[1] and 22.5% for t[2] (R^2^ 0.825, Q^2^ 0.114). The first component (t[1]) was used to differentiate FT2; in turn, the other samples were well separated on the basis of the second principal component (t[2]). In order to understand which variable contributed most to separating the groups, contribution plots were obtained. Two-by-two contribution plots helped to identify the variables responsible for the separation between the two groups (Figure 3A). In particular, ethyl decanoate (*n*. 9) showed the same trend, both in controls and in contaminated samples (Figure 2B and Figure 3B,C), with an increase at early monitoring time and a decrease at Days 5 and 7. Interestingly, an increase in ethyl acetate levels (*n*. 8) resulted at Days 5 and 7 after inoculation (FT5 and FT7) compared to the initial stages of contamination (Figure 3B,C). Higher levels of ethyl acetate (*n*. 8) were observed in the FT5 and FT7 samples compared to controls (Figure 2B and Figure 3C). Furthermore, an increase in 3-methyl butanol (*n*. 15) was observed in response to *F. poae* growth compared to controls. In addition, 1-octen-3-ol and 2,4-dimethyl-1-hepten (*n*. 20, 28) were observed only in FT2 (Figure 3B,C). The production of odorous metabolites, such as 1-octen-3-ol, was already shown to be enhanced as a consequence of other spoilage fungi starting from two days after contamination [21,29].

**Table 1:** GC-MS identification of volatile compounds in the headspace of durum wheat grains in the presence and absence of *F. poae* over seven days.

Identification Number (*n*.)	Compound	Class
1	2-Methylfuran	Aromatics
2	2-Ethyl furan	Aromatics
3	2-Pentylfuran	Aromatics
4	2-Ethyl benzenamine	Aromatics
5	d-Limonene	Terpene
6	4-Ethyl-2-methoxyphenol	Aromatics
7	p-Xylene	Aromatics
8	Ethyl acetate	Esters
9	Ethyl decanoate	Esters
10	Ethyl 9-decenoate	Esters
11	2-Phenylethyl acetate	Esters
12	3-Methylbutanal	Aldehydes
13	Hexanal	Aldehydes
14	Ethanol	Alcohols
15	3-Methyl butanol	Alcohols
16	Propanol	Alcohols
17	2-Methylpropanol	Alcohols
18	2-Pentanol	Alcohols
19	Phenylethyl alcohol	Alcohols
20	1-Octen-3-ol	Alcohols
21	Butanol	Alcohols
22	1-Pentanol	Alcohols
23	Hexanol	Alcohols
24	2-Pentanone	Ketones
25	2-Butanone	Ketones
26	2-Heptanone	Ketones
27	3-Hydroxy-2-butanone	Ketones
28	2,4-Dimethyl-1-hepten	Hydrocarbons
29	Acetic acid	Organic acids

**Figure 4:**
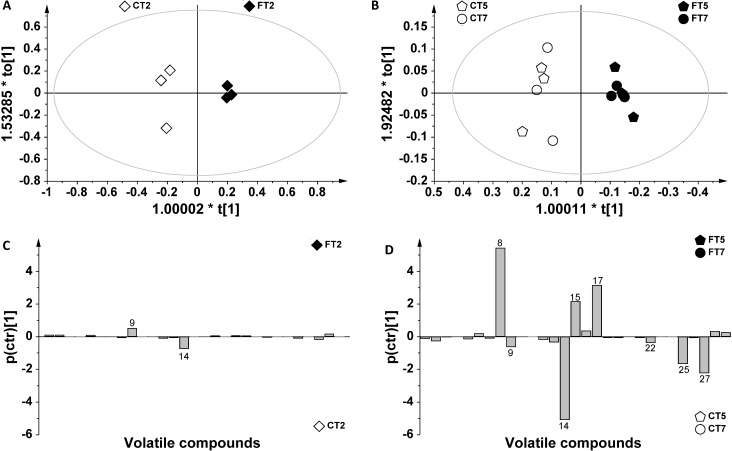
(**A**) Orthogonal partial least squares discriminant analysis (OPLS-DA) score plot of CT2 and FT2 samples (t[1] vs. to[1]); (**B**) OPLS-DA score of CT5/CT7 and FT5/FT7 samples (t[1] vs. to[1]); (**C**) plots of FT2 and CT2 samples showing the contribution of metabolites responsible for differences between infected samples and controls; (**D**) plots of CT5/CT7 and FT5/FT7 showing the contribution of metabolites responsible for differences between infected samples and controls.

Since the largest differences in contaminated *vs.* control samples were observed at Day 2 (FT2/CT2) and at Days 5 and 7, a more detailed orthogonal partial least squares discriminant analysis (OPLS-DA) was performed to enlighten the differences between the groups (Figure 4A,B). In fact, OPLS-DA models improve the separation among samples, maximizing the covariance between the measured data and the responsible variable. The advantage of the OPLS-DA model is that the variation between groups (class separation) can be seen in the first component (t[1]), whereas the variation within groups can be represented in the orthogonal component (to[1]), as described in paragraph 3.4 in the experimental section. In fact, according to recent metabolomic studies, PLS-DA (partial least squares discriminant analysis) and OPLS-DA are the most commonly used methods for the discrimination of samples of different origin [30,31,32]. In this work, the contribution of chromatographic peaks data to group separation was evaluated in the S-line plots (Figure 4C,D).

The OPLS-DA model applied to FT2 and CT2 samples provided a good model. The predictive variation (t[1]) and orthogonal variation (to[1]) corresponded to 58.9% and 40.3%, respectively (Figure 4A). At Day 2, higher levels of ethyl decanoate were found in *F. poae*-infected samples (FT2) with respect to controls (CT2) (Figure 4A). Furthermore, a considerable decrease in ethanol was observed in contaminated grains at Day 2 compared to other FT samples. This result is in line with the metabolic pathway previously suggested for the biosynthesis of trichothecenes [19,33]. In fact, ethanol biosynthesis starts from pyruvate, which is also the precursor for the biosynthesis of trichothecene.

The OPLS-DA analysis applied to FT5/FT7 and CT5/CT7 samples gave good results: the plot of predictive (t[1]) *vs.* orthogonal (to[1]) components showed a good separation of the classes (Figure 4B). Indeed, the predictive variation (t[1]) corresponded to 81.5% of whole data variation, and the uncorrelated variation (orthogonal, to[1]) corresponded to 17.8% (R^2^ 0.993, Q^2^ 0.516). Thus, it was possible to identify the most important volatiles responsible for differences among *F. poae*-contaminated samples with respect to controls (Figure 4D). The samples analyzed at Days 5 and 7 (FT5 and FT7) showed higher levels of ethyl acetate, 3-methyl butanol and 2-methyl propanol (*n*. 8, 15, 17) and lower levels of ethyl decanoate, ethanol, 1-pentanol, 2-butanone and 3-idroxy-2-butanone (*n*. 9, 14, 22, 25, 27). Accordingly, previous works indicated alcohols as major volatiles associated with grain spoilage fungi [22,23], together with a number of other volatile organic compounds, such as ketones and aromatics [20,21]. The decrease in ethanol from the initial analysis time to Days 5 and 7, together with the emergence of acetic acid, might explain the increased levels of ethyl acetate (Figure 3B,C). The reduction in ketones observed over time could be explained as a fungal strategy to eliminate toxic metabolites [34]. On the other hand, 3-methyl butanol and a number of methyl ketones have previously been associated with other food spoilage fungi [35,36,37].

Both PCA and OPLS-DA models had good descriptive ability. In fact, the supervised method, OPLS-DA, efficiently explained the differences between the two subsets of samples (CT and FT at different monitoring times).

**Figure 5:**
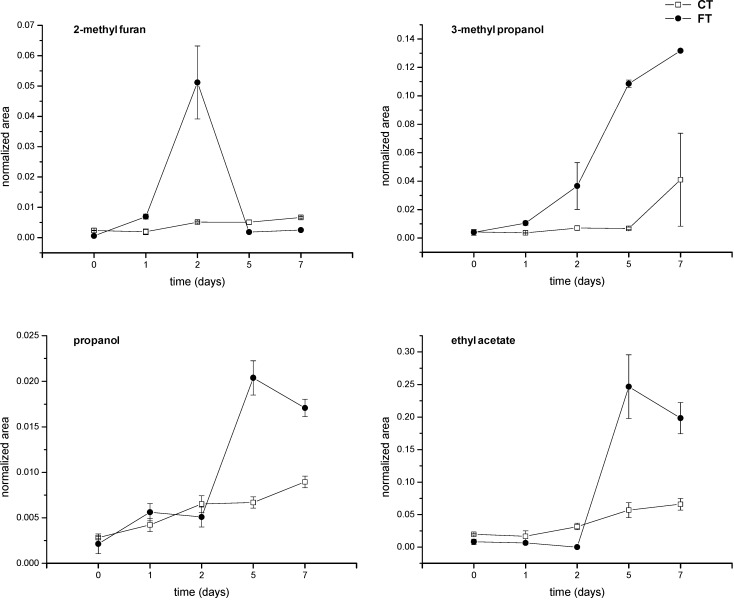
Time course analysis of metabolites detected at different days of analysis in *F. poae*-infected samples (FT) and controls (CT). The data are averages of triplicate analysis (±standard error).

The volatile metabolites that contributed most to discriminating between infected and non-infected samples were considered for time course analysis (Figure 5). The aromatic 2-methylfuran showed an increase in contaminated grains at Day 2 after inoculation (Figure 5). Similar trends were observed for other aromatics (Table 1). Even though the dramatic decrease observed for 2-methyl furan at Days 5 and 7 might be surprising, such a trend has already been observed for other fungi species [21,23]. In general, an increase in aromatic compounds has been found to be associated with cereal grain spoilage. Indeed, 2-methylfuran and 2-penthylfuran have been found to be associated with *Asp. amstelodami*, *Asp. flavus* and *Pen. cyclopium* growth on wheat grains [21] and *Pen. aurantiogriseum* and *Pen. verrucosum* contamination of barley grains [37]. Moreover, p-xylene has been reported in *Asp. niger*, *Asp. flavus*, *Asp. versicolor* and *Asp. candidus* infections [23,38]. In agreement with our findings, the terpene, d-limonene, has been found to be associated with fungal contamination [39].

The time course analysis for 3-methyl propanol and propanol showed that these compounds were produced in higher amounts starting from two days after inoculation (Figure 5). On the other hand, ethyl acetate showed a peak of production at Days 5 and 7, whereas only slight levels of this compound were observed in controls (Figure 5). These findings are comparable with previous evidence concerning *F. culmorum* contaminating wheat grains [21].

In conclusion, volatile metabolites can indicate the presence of different fungi species during grain spoilage. These compounds can be formed during both primary and secondary metabolism from a wide variety of substrates, such as esters, amino acids, fatty acids and keto acids, and the potential exists for distinguishing between fungi species based on characteristic volatile patterns [29,34].

Our study provides the first investigation of volatiles produced by *F. poae* contaminating wheat grains. Even though further analysis will be necessary in order to consider more isolates of *F. poae* and to confirm the present results, this work presents novel insights into the volatile profile of *F. poae* growing on durum wheat grains. The present data appear to confirm previous studies indicating that the volatile profile of fungal-infected samples, rather than individual volatile compounds, could be used to detect wheat grain spoilage [19].

## 3. Experimental Section

### 3.1. Fungal Cultures

ITEM 3258 *F. poae* strain reported to produce trichothecenes [11,40], was grown on solid culture media (1% yeast extract, 2% peptone, 2% glucose, 2% agar) in Petri dishes for 10 days at 25 °C in the dark, washed with sterilized water, and the combined spore suspension was used for inoculation of the medium.

### 3.2. Preparation of Wheat Grains

Durum wheat (*Triticum turgidum* ssp. *durum* (Desf.) Husnot) kernels, moistened to 25% water content and autoclaved at 121 °C for 20 min prior to inoculation, were used as a medium for fungal growth. Incubation was performed in 20-mL glass vials, each filled with 5 g of medium inoculated with 0.5 mL of the aqueous suspension containing approximately 10^7^ conidia mL^−1^. A total of 30 vials were prepared: 15 containing the inoculated grain samples to be analyzed in triplicate on the same day of inoculation (FT0) and 1, 2, 5 and 7 days after inoculation (FT1, FT2, FT5 and FT7, respectively); and 15 controls containing non-contaminated grains to be analyzed in triplicate at the same time as contaminated samples (CT0, CT1, CT2, CT5 and CT7). All vials (FT and CT) were plugged with sterile cotton stoppers and were incubated at 23 °C in the dark for 1, 2, 5 and 7 days. FT0 and CT0 samples were analyzed the same day that they had been prepared. The sterile cotton stoppers were replaced by caps with a Teflon-lined silicon rubber membrane immediately before SPME analysis.

### 3.3. Gas Chromatography and Mass Spectrometry

Headspace analysis was carried out by SHS-SPME/GC/MS in triplicate trials for both contaminated grains and controls. The measures were carried out on the same day of inoculation (FT0) and 1, 2, 5 and 7 days later (FT1, FT2, FT5 and FT7, respectively). The controls were analyzed at the same time as contaminated samples (CT0, CT1, CT2, CT5 and CT7).

The SPME 0.75-μm CAR (Carboxen)-PDMS (polydimethylsiloxane) fibers, purchased from Supelco (Aldrich, Bellefonte, PA, USA), were manually inserted into the sample vial headspace for 30 min at 21.0 (±0.1 °C) in a water bath. They were then desorbed in a split/splitless injector equipped with deactivated SPME glass inserts. Volatiles were analyzed on a 30 m × 250 μm ID × 0.25 μm High Polarity HP INNOWax polyethylene glycol column (Agilent Technologies, Santa Clara, CA, USA) using helium as the gas carrier. A GC system (HP 6890 Series, Agilent Technologies, USA), connected to an HP 5973 mass selective detector (Agilent Technologies, USA), was used. The injections were split 5:1 with a 5-min delay time, and the analysis was carried out at 40 °C for 5 min, then at a rate of 4 °C/min to 150 °C. Subsequently, a temperature of 250 °C was reached at a rate of 15 °C/min. The injector temperature and transfer line were held at 250 °C and at 260 °C, respectively. The helium flow was 1 mL/min. Spectra were produced in an electron impact mode at 70 eV. A mass range of 30–350 amu and a solvent delay time of 4 min were adopted. The threshold used was 150, and the scan rate was 4.45 scans/s. Volatile compounds were identified by comparison with the NIST and other reference IR data available at ChemSpider website [41] and by comparing their spectra with reported fragmentation spectra of authentic standards.

### 3.4. Multivariate Statistical Analysis (MVA) of SPME-GC/MS Data

A data matrix was built from the SPME-GC/MS measurements. For each sample, chromatographic peak data were normalized to the total sum of peak areas, in order to make variables comparable to each other. Multivariate statistical analysis and graphics were obtained using Simca-P version 13.0.2 (Umetrics, Sweden), and PCA and OPLS-DA were used [42]. PCA allowed the remapping of the original dataset in a new multivariate coordinate space (score plot) in which the dimensions were ordered by the decreasing explained variance in the data. The score plot was defined by the principal components, namely t[*n*], with *n* indicating the number of the considered components (in our case *n* = 1, 2) and it showed the samples displayed as a set of scores that highlighted clustering and outliers. In a separate graph (loading plot) a set of loadings (p[*n*]) were displayed to emphasize the influence of input variables on the t[*n*] components. Furthermore, OPLS-DA focused the predictive information into one component, thus facilitating the interpretation of the spectral data. Component t[1] was the predictive component and displayed the between-class variation of the samples. Component to[1] was the Y-orthogonal component and modeled the within group (within-class) variation. The robustness and predictive ability of the OPLS-DA models for discrimination purposes were tested by cross-validation [43,44].
